# A Practical Manual of Diseases of the Skin

**Published:** 1892-05

**Authors:** 


A Practical Manual of Diseases of the Skin. By George H. Rohe, M. D., Professor of Materia Medica, Therapeutics, and Hygiene, and formerly Professor of Dermatology in the College of Physicians and Surgeons, Baltimore, etc., etc. Assisted by J. Williams Lord, A. B., M. D., Lecturer on Dermatology and Bandaging in the College of Physicians and Surgeons; Assistant Physician to the Skin Department in the Dispensary of Johns Hopkins Hospital. No. 13 in the *Physicians' and Students' Ready-Reference Series*. In one neat 12mo volume, 303 pages. Extra cloth. Price, $1.25 net. Philadelphia: The F. A. Davis Co., Publishers, 1231 Filbert street.

There is no more difficult class of diseases to understand or to control, in so far as the general practitioner is concerned, than those which involve the skin when they are of a chronic nature. The dermatologist masters the science of the specialty with considerable ease and much perfection, but the general practitioner continually prays to be delivered from the treatment of chronic skin diseases. Dr. Rohé has had a large experience both as a specialist and as a general practitioner, and writes from a standpoint that makes his book of great value to the professional world. In the preparation of the book, the distinguished author has also had the valuable assistance of Dr. Lord, his clinical aid at the College of Physicians and Surgeons in Baltimore, and between the two they have contrived the most valuable little manual that we are familiar with. We advise every physician who lives at a distance from the great centers to obtain this book and read it carefully. It will give him much additional light on what has oftentimes proven a dark subject to him, and enable him to conduct his cases with much more satisfaction to himself and his patients. He will cure many of the milder diseases, will early recognize the intractable forms, and will speedily send them to a dermatologist who will aid him in his dilemma.

